# Long-term outcome after allogeneic stem cell transplantation in multiple myeloma

**DOI:** 10.1007/s00277-021-04514-y

**Published:** 2021-04-17

**Authors:** Sini Luoma, Raija Silvennoinen, Auvo Rauhala, Riitta Niittyvuopio, Eeva Martelin, Vesa Lindström, Jouni Heiskanen, Liisa Volin, Tapani Ruutu, Anne Nihtinen

**Affiliations:** 1 Comprehensive Cancer Center, Department of Hematology, Helsinki University Hospital and University of Helsinki, Haartmaninkatu 4, P.O. Box 372, 00290 Helsinki, Finland; 2 Department of Medicine, Kuopio University Hospital, Kuopio, Finland; 3 Faculty of Education and Welfare Studies, Åbo Akademi University, Vaasa, Finland; 4 Vaasa Central Hospital, Vaasa, Finland; 5 Clinical Research Institute, Helsinki University Hospital and University of Helsinki, Helsinki, Finland; 6 Department of Internal Medicine, North Carelia Central Hospital, Joensuu, Finland

**Keywords:** Multiple myeloma, Allogeneic stem cell transplantation, Survival, Conditioning, Graft-versus-host disease, Post-relapse survival

## Abstract

The role of allogeneic hematopoietic stem cell transplantation (allo-SCT) in multiple myeloma is controversial. We analyzed the results of 205 patients transplanted in one center during 2000–2017. Transplantation was performed on 75 patients without a previous autologous SCT (upfront-allo), on 74 as tandem transplant (auto-allo), and on 56 patients after relapse. Median overall survival (OS) was 9.9 years for upfront-allo, 11.2 years for auto-allo, and 3.9 years for the relapse group (*p* = 0.015). Progression-free survival (PFS) was 2.4, 2.4, and 0.9 years, respectively (*p* < 0.001). Non-relapse mortality at 5 years was 8% overall, with no significant difference between the groups. Post-relapse survival was 4.1 years for upfront-allo and auto-allo, and 2.6 years for the relapse group (*p* = 0.066). Survival of high-risk patients was reduced. In multivariate analysis, the auto-allo group had improved OS and chronic graft-versus-host disease was advantageous in terms of PFS, OS, and relapse incidence. Late relapses occurred in all groups. Allo-SCT resulted in long-term survival in a small subgroup of patients. Our results indicate that auto-allo-SCT is feasible and could be considered for younger patients in the upfront setting.

## Introduction

Allogeneic stem cell transplantation (allo-SCT) is thus far the only potentially curative treatment approach in multiple myeloma (MM), but only a fraction of patients are eligible for it. The use of allo-SCT is limited by transplant-related mortality (TRM) that can rise to 41% with myeloablative conditioning (MAC) [1]. With reduced-intensity conditioning (RIC), the TRM is lower (10–15%), but the risk of relapse is higher than that with MAC [2–4]. Reduced toxicity conditioning may offer survival outcomes equal to that of MAC but a more acceptable TRM [5, 6].

The role of allo-SCT in the treatment of MM is under debate. This is based on the contradicting results of previous studies over the survival advantage allo-SCT may offer over autologous stem cell transplantation (ASCT), and the disappointing relapse rate after allo-SCT [2–4, 7–9]. However, a proportion of patients seem to remain in long-term remission [3, 6, 8]. It is also unclear, whether the graft-versus-myeloma effect of allo-SCT [10] can overcome the poor prognosis of high-risk (HR) patients [11–16].

Allo-SCT can be considered in MM as the first-line treatment, with or without a previous ASCT, or in relapsed disease as a salvage treatment. Current guidelines recommend considering allo-SCT only after disease relapse or as a part of a clinical trial [17–19]. Still, allo-SCTs are performed outside of clinical trials in considerable amounts [20].

In this retrospective single-center study, we report the results of allo-SCT performed in three different settings: upfront without a previous ASCT, after ASCT as a tandem transplant (auto-allo), and after relapse. Our aim was to determine the outcomes of these transplant strategies. Our second point of interest was the outcome of patients with HR cytogenetics.

## Methods

### Study design and population

We included all patients with MM who underwent allo-SCT between January 2000 and December 2017 in Helsinki University Hospital Stem Cell Transplantation Unit. Primary plasma cell leukemia was an exclusion criterion. The data was collected from the European Group for Blood and Marrow Transplantation (EBMT) database. All patients provided written informed consent for the EBMT reporting. Data not included in the EBMT reports was collected from the medical records as approved by our institutional review board. This study was conducted according to the Declaration of Helsinki, International Conference of Harmonization and Guidelines for Good Clinical Practice.

Helsinki University Hospital is one of the two Finnish allogeneic stem cell transplantation centers. Patients are referred to us from all parts of Finland except for the southwest district. The standard operating procedures of our transplantation unit have recommended allo-SCT to be considered in an upfront setting for young patients, mostly under 50–55 years of age with extramedullary disease or advanced bone disease. The choice of transplanting the patients with or without a previous ASCT was based on clinical decision-making.

### Definitions and endpoints

For the purpose of this analysis, patients were divided into three subgroups, referred to here as “therapy groups”: (1) “upfront-allo” group who received allo-SCT in first line without a previous ASCT, (2) “auto-allo” group, where allo-SCT was performed as a preplanned tandem therapy after ASCT, and (3) “relapse” group who received allo-SCT after at least one relapse, with or without a previous ASCT.

HR cytogenetics was defined as the presence of del17p, t(4;14), or t(14;16) [21]. In 2000–2002, chromosome analysis was performed with G band karyotyping. Since 2003, the use of fluorescence in situ hybridization started to increase, with a widening array of probes. In 2010, the incorporation of CD138+ plasma cell selection method further improved the sensitivity of analyses. Therefore, data of HR cytogenetics was available to us sporadically in 2003–2009 and systematically from the year 2010 on.

Conditioning regimen intensities and graft-versus-host disease (GVHD) were graded according to previously published criteria [22–25]. The International Myeloma Working Group (IMWG) criteria were used to define MM disease status and relapse [26].

The data cutoff was 20 December 2019. The primary endpoint was overall survival (OS) according to the therapy group. Secondary endpoints included progression-free survival (PFS), relapse incidence (RI), post-relapse survival (PRS), non-relapse mortality (NRM), and GVHD- and relapse-free survival (GRFS). GRFS was defined as being alive with neither grade 3-4 acute GVHD (aGVHD), systemic therapy-requiring chronic GVHD (cGVHD), nor disease relapse at any time point [27].

### Statistical analysis

Statistical analyses were performed according to the EBMT Statistical Guidelines [28]. Patient-, disease-, and transplant-related variables of the therapy groups were compared using Pearson’s chi-square test or Fisher’s exact test for categorical variables, and Mann-Whitney test or Kruskal-Wallis test for continuous variables. The probabilities of OS, PFS, PRS, and GRFS were calculated using the Kaplan-Meier method and the log-rank test for univariate comparisons. Acute and chronic GVHD, RI, and NRM were calculated by using the cumulative incidence (CI) estimator to accommodate competing risks. Gray’s test was used for between-group tests. For NRM, relapse was the competing risk, and for RI, the competing risk was death without relapse. Multivariate analyses for OS and PFS were performed using Cox proportional hazards regression model and for RI by the Fine-Gray method. Gender, age, and Karnofsky performance status of the patient, International Staging System (ISS) stage, presence of extramedullary disease, number of treatment lines and disease status before allo-SCT, therapy group, year of the allo-SCT, conditioning regimen intensity, graft type, donor source, donor/recipient gender mismatch and CMV status, and presence of GVHD were tested in univariate analyses. Variables with *p*-values < 0.3 were taken into multivariate analyses. *p*-values are two-sided. Statistical analyses were performed with the SPSS 25 (SPSS inc./IBM, Armonk, NY, USA) and R version 3.6.2. (R Core Team. 2019) [29] software packages.

## Results

### Patient and transplant characteristics

Baseline characteristics are summarized in Table 1. Eighteen patients (9%) participated in a clinical study; 15 in the EBMT-NMAM2000 study [4] and three in a treosulfan-based conditioning regimen study [30].

**Table 1:** Patient, disease, and transplant characteristics

Characteristics	Overall	Upfront-allo	Auto-allo	Relapse	*P*	Missing
*N* (%)	205 (100)	75 (37)	74 (36)	56 (27)		
Age, years, median (range)	51.7 (26.3–66.1)	47.4 (26.3–59.7)	53.2 (30.7–65.1)	56.1 (41.9–66.1)	< 0.001	
< 50	83 (40)	47 (63)	24 (32)	12 (21)		
50–60	98 (48)	28 (37)	42 (57)	28 (50)		
> 60	24 (12)	0 (0)	8 (11)	16 (29)		
Gender male/female	105/100 (51/49)	42/33 (56/44)	34/40 (46/54)	29/27 (52/48)	0.468	
Myeloma subtype					0.092	
IgG	121 (59)	45 (60)	47 (64)	29 (52)		
IgA	33 (16)	8 (11)	9 (12)	16 (28)		
IgD	5 (2)	4 (5)	1 (1)	0 (0)		
Light chain	43 (21)	17 (23)	15 (20)	11 (20)		
Nonsecretory	3 (2)	1 (1)	2 (3)	0 (0)		
ISS stage					0.385	30 (14)
I	71 (35)	31 (41)	21 (28)	19 (34)		
II	69 (34)	22 (29)	31 (42)	16 (29)		
III	35 (17)	13 (17)	15 (20)	7 (13)		
Cytogenetic risk					0.054	118 (58)
Standard	56 (27)	30 (40)	13 (18)	13 (23)		
Higha	31 (15)	9 (12)	14 (19)	8 (14)		
Extramedullary diseaseb	63 (31)	35 (47)	13 (18)	15 (27)	0.001	7 (3)
Number of pre-allo-SCT treatment lines					< 0.001	1
1	74 (36)	31 (41)	43 (58)	0 (0)		
2	67 (33)	25 (33)	23 (31)	19 (34)		
3	44 (22)	14 (19)	7 (10)	23 (42)		
≥ 4	19 (9)	5 (7)	1 (1)	13 (23)		
Induction treatment					< 0.001	1
AD/VAD	79 (39)	17 (23)	40 (54)	22 (39)		
VCD	46 (23)	19 (25)	21 (28)	6 (11)		
BorDex	27 (13)	11 (15)	5 (7)	11 (20)		
Thalidomide-based combination	27 (13)	16 (21)	2 (3)	9 (16)		
RVD	13 (6)	6 (8)	3 (4)	4 (7)		
MP	2 (1)	0 (0)	0 (0)	2 (4)		
Otherc	10 (5)	6 (8)	3 (4)	1 (2)		
Novel drugs prior to allo-SCT					< 0.001	
IMID	110 (54)	44 (59)	22 (30)	44 (79)		
PI	128 (62)	55 (73)	37 (50)	36 (64)		
IMID and PI	82 (40)	35 (47)	18 (24)	29 (52)		
None	49 (24)	11 (15)	33 (45)	5 (9)		
Disease status prior to allo-SCT					0.093	1
sCR/CR	42 (20)	10 (14)	22 (30)	10 (18)		
VGPR/PR	149 (73)	60 (80)	49 (66)	40 (71)		
MR/SD	3 (1)	1 (1)	0 (0)	2 (4)		
PD	10 (5)	4 (5)	2 (3)	4 (7)		
Time between diagnosis and 1st auto-SCT, days, median (range)	206 (113–2643)		195 (119–2643)	219 (113–2300)	0.092	
Time between last auto-SCT and allo-SCT, days, median (range)d	205 (63–4459)		161 (63–391)	750 (113–4459)	< 0.001	
Time between diagnosis and allo-SCT, days, median (range)	377 (137–5104)	261 (137–1721)	372 (218–2776)	1145 (246–5104)	< 0.001	
Karnofsky performance status ≥ 80	199 (97)	70 (93)	73 (99)	56 (100)	0.192	1
Donor source					0.030	
Sibling	100 (49)	33 (44)	45 (61)	22 (39)		
HLA matched	99 (48)	33 (44)	45 (61)	21 (38)		
9/10 HLA matched	1 (1)	0 (0)	0 (0)	1 (1)		
MUD	105 (51)	42 (56)	29 (39)	34 (61)		
10/10 HLA matched	89 (43)	34 (45)	23 (31)	32 (57)		
≤ 9/10 HLA matched	16 (8)	8 (11)	6 (8)	2 (4)		
Donor/recipient gender					0.520	
M/M	73 (36)	32 (43)	24 (32)	17 (30)		
M/F	52 (25)	15 (20)	23 (31)	14 (25)		
F/M	32 (16)	10 (13)	10 (14)	12 (22)		
F/F	48 (23)	18 (24)	17 (23)	13 (23)		
Graft type					< 0.001	
BM	39 (19)	28 (37)	4 (5)	7 (12)		
PB	166 (81)	47 (63)	70 (95)	49 (88)		
Conditioning regimen					< 0.001	
MACe	88 (43)	73 (97)	4 (5)	11 (20)		
CyTBI	55 (27)	53 (70)	0 (0)	2 (4)		
Treo14	33 (16)	20 (27)	4 (5)	9 (16)		
RICf	117 (57)	2 (3)	70 (95)	45 (80)		
FluTBI	52 (25)	1 (1)	42 (57)	9 (16)		
Treo-RIC	65 (32)	1 (1)	28 (38)	36 (64)		
Received ATG	119 (58)	35 (47)	27 (36)	23 (41)	0.409	1
GVHD prophylaxis					< 0.001	
CSA + short course of MTX	128 (63)	50 (67)	32 (43)	46 (82)		
CSA + short course of MTX + MP	25 (12)	24 (32)	0 (0)	1 (2)		
CSA + MMF	52 (25)	1 (1)	42 (57)	9 (16)		

^a^Defined by the presence of del17p (any percentage), t(4;14), or t(14;16)

^b^The presence of plasma cells or plasmacytomas outside the bone marrow

^c^4 patients received dexamethasone monotherapy; 3 bortezomib, doxorubicin, and dexamethasone; 2 cyclophosphamide-dexamethasone; 1 cyclophosphamide, doxorubicin, and dexamethasone

^d^11 patients received two auto-SCTs

^e^MAC regimens included CyTBI (cyclophosphamide 60 mg/kg for 2 days and total body irradiation 12 Gy) and Treo14 (treosulfan 14 g/m^2^ for 3 days and fludarabine 30 mg/m^2^ for 5 days)

^f^RIC regimens included FluTBI (fludarabine 30 mg/m^2^/3 days and total body irradiation 2 Gy) and Treo-RIC (treosulfan 10–12 g/m^2^ for 3 days and flurarabine 30 mg/m^2^ for 5 days)

*Upfront-allo* allo-SCT performed first line without a previous auto-SCT, *auto-allo* allo-SCT performed after auto-SCT in first line, *relapse* allo-SCT performed after relapse, *ISS* International Staging System, *allo-SCT* allogeneic hematopoietic stem cell transplantation, *AD* doxorubicin and dexamethasone, *VAD* vincristine, doxorubicin, and dexamethasone, *VCD* bortezomib, cyclophosphamide, and dexamethasone, *BorDex* bortezomib and dexamethasone, *RVD* bortezomib, lenalidomide, and dexamethasone, *MP* melphalan and prednisone, *IMID* immunomodulatory drug, *PI* proteasome inhibitor, *sCR* stringent complete response, *CR* complete response, *VGPR* very good partial response, *PR* partial response, *MR* minimal response, *SD* stable disease, *PD* progressive disease, *auto-SCT* autologous stem cell transplantation, *HLA* human leucocyte antigen, *MUD* matched unrelated donor, *BM* bone marrow, *PB* peripheral blood, *MAC* myeloablative conditioning, *RIC* reduced-intensity conditioning, *ATG* antithymocyte globulin, *GVHD* graft-versus-host disease, *CSA* cyclosporine, *MTX* methotrexate, *MP* methylprednisolone, *MMF* mycophenolate mofetil

Gender, MM subtype, ISS stage, and HR cytogenetics were balanced across the groups. The patients in the upfront-allo group were younger, had more extramedullary disease, and received bone marrow grafts more often than patients in the other two groups. The patients in the relapse group were older than those in the other groups. Eighty-nine (43%) transplantations were performed in 2000–2007 and 116 (57%) in 2008–2017. The most common induction therapy was doxorubicin and dexamethasone, with or without vincristine (AD/VAD), mostly given in 2000–2006. Thalidomide-based combinations were used in first line in 2006–2013, bortezomib and dexamethasone (BorDex) from 2007 onwards, and bortezomib in combination with cyclophosphamide and dexamethasone (VCD) or lenalidomide and dexamethasone (RVD) from 2012 onwards. The majority of the patients had received treatment with immunomodulators or proteasome inhibitors before the allo-SCT, with 62% of the patients being treated with bortezomib and 28% with thalidomide. Lenalidomide was reimbursed in Finland in 2010 and 28% of the patients had received it before the transplantation. Four different conditioning regimens were used. Maintenance treatment was not routinely used after allo-SCT.

The median number of CD34+ cells in peripheral blood grafts was 6.1 (range, 1.6–15.3) × 10^6^/kg. In bone marrow grafts, the median total nucleated cell count of 25 grafts was 2.8 (range, 1.6–5.6) × 10^8^/kg, and the median number of mononuclear cells of 14 grafts 0.5 (range, 0.2–1.4) × 10^8^/kg.

### Response and GVHD

Overall response rate (ORR) was 86%, with 51% of the patients achieving sCR or CR. Out of 164 patients with available data on chimerism, 157 (96%) achieved full donor chimerism. Cumulative incidence of aGVHD grades 2–4 at day 100 was 24%. Chronic GVHD occurred in 62% of the patients and it was extensive in 47% (Table 2).

**Table 2:** Response and transplant-related toxicity

Variable	Overall	Upfront-allo	Auto-allo	Relapse	*P*	Missing
*N* (%)	205 (100)	75 (37)	74 (36)	56 (27)		
Median engraftmenta, days (range)	17 (1–59)	16 (1–31)	17 (1–47)	17 (1–59)	0.390	
Overall response rate (≥ PR)	177 (86)	68 (91)	67 (91)	42 (75)	0.012	3 (1)
Best response after allo-SCT					0.072	3 (1)
sCR	26 (13)	11 (15)	13 (18)	2 (4)		
CR	77 (38)	30 (40)	32 (43)	15 (27)		
VGPR	49 (24)	19 (25)	15 (20)	15 (27)		
PR	25 (12)	8 (11)	7 (9)	10 (18)		
MR	2 (1)	1 (1)	0 (0)	1 (2)		
SD	3 (1)	0 (0)	1 (1)	2 (4)		
PD	20 (10)	5 (7)	5 (7)	10 (18)		
aGVHD grades 2–4b (%, 95% CI)	24% (18–30%)	21% (12–31%)	31% (20–42%)	20% (9–30%)	0.330	
2	12% (7–16%)	8% (2–14%)	15% (7–23%)	13% (4–21%)	0.431	
3	10% (6–14%)	13% (6–21%)	11% (4–18%)	5% (0–11%)	0.369	
4	2% (0–5%)	0	5% (0–11%)	2% (0–5%)	0.094	
cGVHD at 5 years (%, 95% CI)	62% (55–69%)	58% (46–69%)	74% (64–84%)	52% (38–65%)	0.008	
ext. cGVHD at 5 years (%, 95% CI)	47% (40–54%)	41% (30–53%)	58% (47–69%)	39% (26–52%)	0.031	

^a^Defined as the first of three consecutive days with an absolute neutrophil count > 0.5 × 10^9^/l. Two engraftment failures, 1 due to PD and one due to early death

^b^Cumulative incidence at 100 days post-transplant

*Upfront-allo* allo-SCT performed first line without a previous auto-SCT, *auto-allo* allo-SCT performed after auto-SCT in first line, *relapse* allo-SCT performed after relapse, *allo-SCT* allogeneic hematopoietic stem cell transplantation, *sCR* stringent complete response, *CR* complete response, *VGPR* very good partial response, *PR* partial response, *MR* minimal response, *SD* stable disease, *PD* progressive disease, *aGVHD* acute graft-versus-host disease, *cGVHD* chronic graft-versus-host disease, *ext. cGVHD* extensive chronic graft-versus-host disease

### Multivariate analysis for factors affecting survival and relapse incidence

Table 3 shows the results of the multivariate analysis. Independent factors for longer OS were female gender, lower ISS stage, MAC, auto-allo therapy group, and cGVHD. Acute GVHD grade ≥ 2 reduced OS. Predictive factors for reduced PFS were higher ISS stage, relapse group, and aGVHD grade ≥ 2. Chronic GVHD predicted longer PFS. Chronic GVHD, age ≤ 55 years, Karnofsky performance scale ≥ 80, lower number of prior treatment lines, and not having extramedullary disease were associated with lower RI.

**Table 3:** Results of multivariate analysis

Variable	OS		PFS		RI	
	HR (95% CI)	*P*	HR (95% CI)	*P*	HR (95% CI)	*P*
Age		*NS*		*NS*		
≤ 55					Reference	
≥ 56					1.85 (1.28–2.68)	**0.001**
Gender						
Male	Reference					
Female	0.57 (0.37–0.89)	**0.014**				
ISS stage						
I	Reference		Reference			
II	1.88 (1.12–3.15)	**0.018**	1.22 (0.81–1.85)	0.348		
III	3.14 (1.77–5.59)	**< 0.001**	1.87 (1.16–3.02)	**0.010**		
No. of prior therapy lines		*NS*		*NS*		
1					Reference	
≥ 2					1.73 (1.22–2.46)	**0.002**
Extramedullary disease				*NS*		
No					Reference	
Yes					1.59 (1.13–2.26)	**0.009**
Karnofsky performance status						
≥80					Reference	
< 80					4.16 (2.49–6.95)	< **0.001**
Conditioning regimen				*NS*		
MAC	Reference					
RIC	3.85 (1.25–11.88)	**0.019**				
Therapy group						*NS*
Upfront-allo	Reference		Reference			
Auto-allo	0.25 (0.075–0.81)	**0.022**	0.93 (0.60–1.46)	0.758		
Relapse	0.63 (0.20–1.99)	0.432	2.25 (1.43–3.55)	**< 0.001**		
aGVHD ≥ gr.2						*NS*
Yes	Reference		Reference			
No	0.27 (0.17–0.45)	**< 0.001**	0.42 (0.28–0.65)	**< 0.001**		
cGVHD						
Yes	Reference		Reference		Reference	
No	2.62 (1.67–4.12)	**< 0.001**	2.30 (1.57–3.36)	**< 0.001**	1.61 (1.13–2.29)	**0.008**

*OS* overall survival, *PFS* progression-free survival, *RI* relapse incidence, *ISS* International Staging System, *MAC* myeloablative conditioning, *RIC* reduced intensity conditioning, *Upfront-allo* allo-SCT performed first line without a previous auto-SCT, *auto-allo* allo-SCT performed after auto-SCT in first line, *relapse* allo-SCT performed after relapse, *aGVHD* acute graft-versus-host disease, *cGVHD* chronic graft-versus-host disease, *NS* not significant

Bold values indicate statistical significance at *P* < 0.05

To further examine the effect of cGVHD on survival, a landmark analysis was done with patients (*n* = 199) who had survived at least for 100 days after the transplantation. The OS difference remained significant. In this analysis, median OS was 11.4 years (95% CI, 6.9–15.8) with and 3.7 years (95% CI, 2.2–5.1) without cGVHD (*p* < 0.001).

### Survival outcomes by therapy groups

**Fig. 1:**
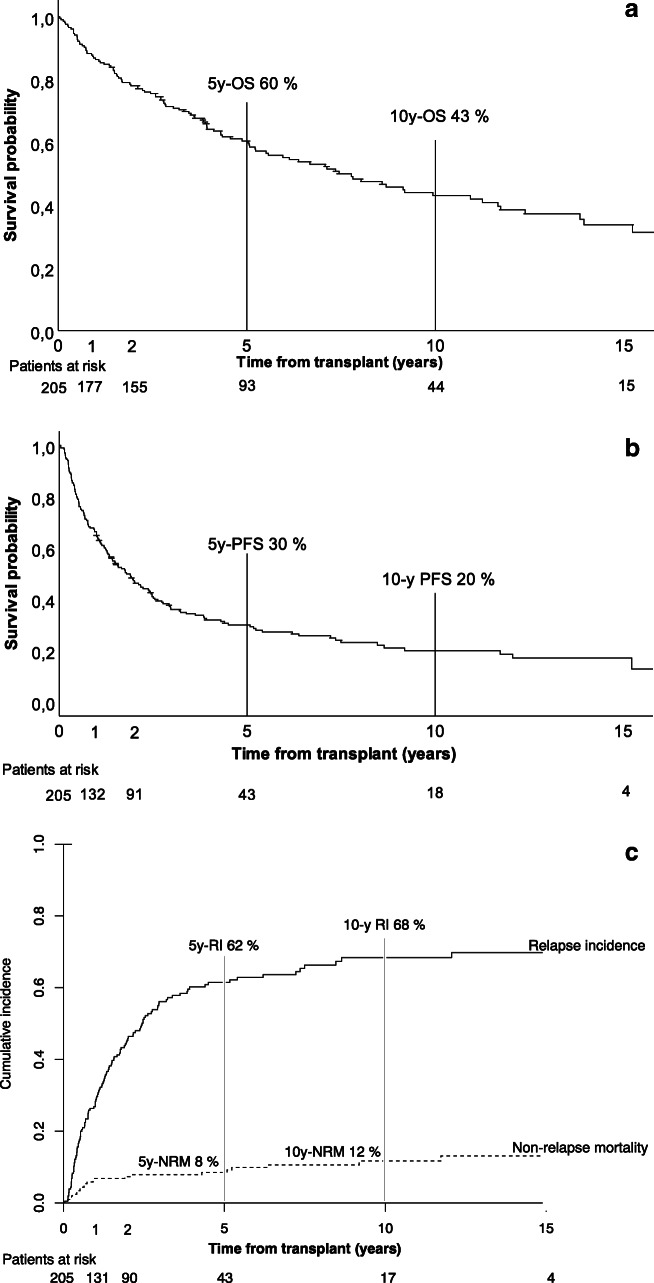
Overall survival (**a**), progression-free survival (**b**), and relapse incidence and non-relapse mortality (**c**) in the whole cohort, *n* = 205

**Fig. 2:**
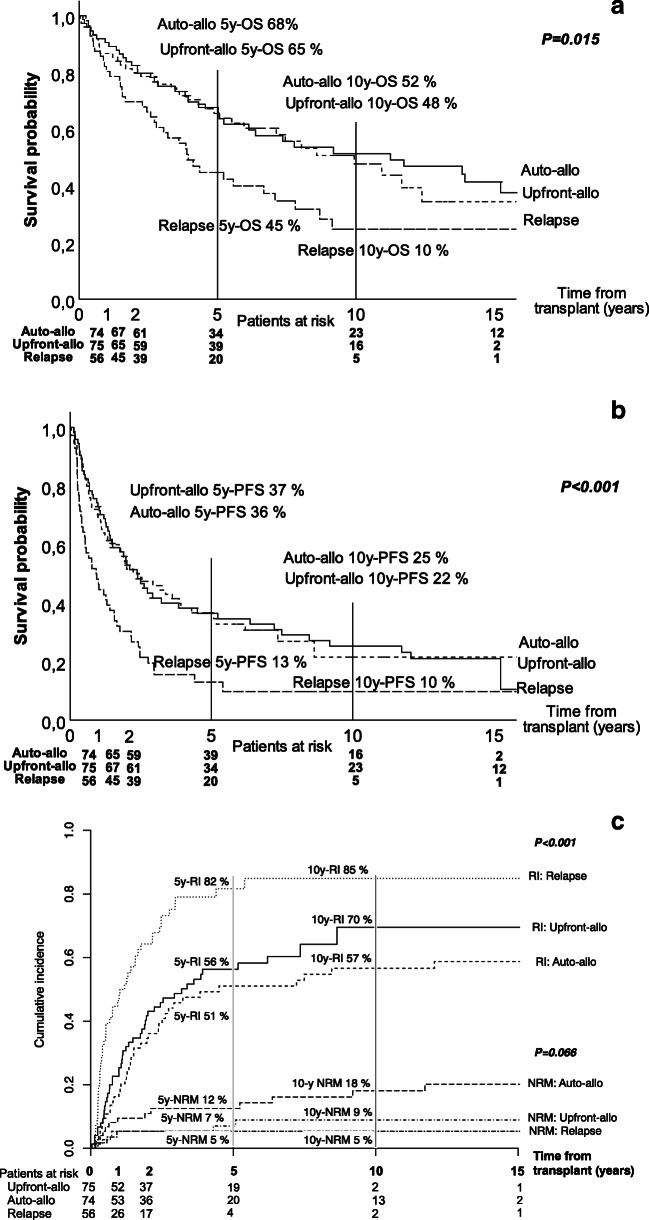
Overall survival (**a**), progression-free survival (**b**), and relapse incidence and non-relapse mortality (**c**) according to the therapy group

With a median follow-up of 4.3 years (range, 0–18.4) for the entire cohort and 5.2 years (range 1.1–18.4) for patients surviving over 1 year, the median OS was 7.4 years (95% CI: 5.1–9.8) (Fig. 1a). By therapy groups, the median OS were 9.9 years (95% CI: 6.0–13.9), 11.2 years (95% CI: 3.8–18.7), and 3.9 years (95% CI: 3.0–4.9) (*p* = 0.015) for upfront-allo, auto-allo, and relapse groups, respectively (Fig. 2a).

The median PFS of the entire cohort was 1.8 years (95% CI: 1.3–2.3) with a median follow-up of 1.6 years (range, 0–17.2) (Fig. 1b). By therapy groups, the median PFS were 2.4 years (95% CI: 1.1–3.8), 2.4 years (95% CI: 1.6–3.1), and 0.9 years (95% CI: 0.5–1.4) (*p* < 0.001) for upfront-allo, auto-allo, and relapse groups, respectively (Fig. 2b). The median OS and PFS of the eighteen patients participating in the clinical studies did not differ from other patients in our study.

When the disease status in the auto-allo group before allo-SCT was sCR or CR (*n* = 22), median OS was not reached, compared with median OS of 11.2 years (95% CI, 4.5–18.0) with VGPR or PR (*n* = 49), and 6.1 years (95% CI not calculated, *n* = 2) with disease status less than PR (*p* = 0.627). This effect was not seen in the other two therapy groups. Disease status before first ASCT did not affect survival outcomes in auto-allo or relapse groups. The number of previous treatment lines did not significantly affect OS in any therapy group. In the relapse group, median OS were 3.9 years (95% CI, 1.9–5.8) after two, 6.7 years (95% CI, 2.3–11.1) after three, and 2.7 years (95% CI, 0–6.4) after four or more previous lines (*p* = 0.363). The median PFS in the relapse group were 0.8 years (95% CI, 0–1.5) after two, 1.3 years (95% CI, 0.3–2.3) after three, and 0.6 years (95% CI, 0–1.3) after four or more previous lines (*p* = 0.482). There was no difference in OS in any therapy group according to whether the patients had received treatment with immunomodulators or proteasome inhibitors as induction therapy or in later line before the allo-SCT or not.

Patients with cGVHD had median OS of 11.7 years (95% CI, 7.2–16.1) compared with 3.6 years (95% CI, 2.3–5.0) (*p* < 0.001) for those without it. Median PFS was 2.6 years (95% CI, 1.9–3.3) for patients with and 1.0 years (95% CI, 0.5–1.5) (*p* < 0.001) for patients without cGVHD. Median GRFS was 0.6 years (95% CI, 0.5–0.7) for the entire cohort, with 18% at 2 years and 10% at 5 years. The cumulative incidence of NRM was 8% at 5 years overall (Fig. 1c) and it was the highest (12%) in the auto-allo group (Fig. 2c).

### Relapse incidence and post-relapse survival by therapy groups

Relapse incidence for the entire cohort was 62% at five and 68% at ten years (Fig. 1c). By therapy groups, RI at 5 years were 56% for upfront-allo, 51% for auto-allo, and 82% for relapse (*p* < 0.001) (Fig. 2c).

The median PRS for all patients was 3.5 years (95% CI, 2.7–4.2); 68% at 2, 38% at 5, and 25% at 10 years. For upfront-allo, auto-allo, and relapse groups, the median PRS were 4.1 years (95% CI, 0.5–8.1), 4.1 years (95% CI, 1.9–6.3), and 2.6 years (95% CI, 2.0–3.2), respectively (*p* = 0.066). In patients with cGVHD, the median PRS was significantly longer with 6.5 years (95% CI, 3.2–9.9), compared with 2.1 years (95% CI, 1.1–3.0) in others (*p* < 0.001).

Forty-nine patients were treated with donor lymphocyte infusion (DLI), nine of them prophylactically. When comparing patients who received DLI for treatment of PD with others, there was no difference in PRS (data not shown).

### Patients with high-risk disease

A complete cytogenetic analysis was performed in 87 (42%) patients and 31 (15%) had HR features. Their median OS was reduced compared with standard risk (SR) patients (Table 4). There was no difference in NRM between patients with HR and SR cytogenetics. Median PRS was 1.1 years (95 % CI, 0.5–1.7) for patients with HR cytogenetics (*n* = 20) and 4.1 years (95 % CI, 2.1–6.0) for SR cytogenetics (*n* = 36) (*p* = 0.002). The information on the ISS stage was available for 175, the R-ISS stage for 82, and IMWG risk stratification for 86 patients; and all correlated with survival outcomes (Table 4).

**Table 4:** Survival of patients by risk groups

Characteristics	*N* (%)	OS, median, years (95% CI)	*P*	PFS, median, years (95% CI)	*P*	Missing, *N* (%)
Cytogenetic risk			0.039		0.429	118 (58)
Standard	56 (27)	NR		2.0 (1.0–2.9)		
Higha	31 (15)	2.9 (1.1–4.6)		1.2 (0.7–1.8)		
ISS stage			0.002		0.044	30 (14)
I	71 (35)	16.1 (7.0–25.3)		2.4 (1.4–3.4)		
II	69 (34)	6.4 (3.5–9.2)		1.8 (0.8–2.7)		
III	35 (17)	2.5 (0.9–4.0)		0.8 (0.0–1.7)		
R-ISS stage			0.008		0.006	123 (60)
I	13 (6)	NR		2.5 (0.0–5.8)		
II	55 (27)	8.0 (1.4–14.7)		1.7 (1.0–2.5)		
III	14 (7)	1.7 (0.7–2.7)		0.4 (0.1–0.9)		
IMWG risk			0.048		0.047	119 (58)
Low	29 (14)	NR		2.0 (0.1–3.8)		
Standard	35 (17)	NR		2.5 (1.5–3.4)		
High	22 (11)	2.6 (1.1–4.1)		0.6 (0.2–1.0)		

^a^15 patients (7%) had del17p, 22 (11%) t(4;14), and two (1%) t(14;16)

*OS* overall survival, *PFS* progression-free survival, *ISS* International Staging System, *R-ISS* Revised International Staging System, *IMWG risk* International Myeloma Working Group Risk Stratification, *NR* not reached

### Outcomes by conditioning regimens

Patients transplanted with MAC had longer median OS of 10.9 years (95% CI, 7.0–14.9) compared with 5.2 years (95% CI, 2.5–7.9) in patients receiving transplant after RIC (*p* = 0.027). There was no difference in PFS, RI, or NRM between MAC and RIC.

When comparing the four different conditioning regimens used (Table 1), the median OS was 11.7 years (95% CI, 6.3–17.0), 9.9 years (95% CI, 5.9–14.0), 6.4 years (95% CI, 2.1–10.7), and 4.1 years (95% CI, 1.0–7.2) (*p* = 0.042); and the median PFS 3.4 years (95% CI, 1.2–5.5), 2.2 years (95% CI, 1.7–2.7), 1.6 years (95% CI, 0.7–2.4), and 1.2 years (95% CI, 0.7–1.7) (*p* = 0.009) after conditioning with CyTBI, Treo14, FluTBI, and Treo-RIC, respectively. PFS at 5 years were 43% for CyTBI, 18% for Treo14, 35% for FluTBI, and 19% for Treo-RIC conditioning (*p* = 0.025). NRM was highest for FluTBI regimen with 17% at 5 years, whereas it was 7% with CyTBI, 3% with Treo14, and 5% with Treo-RIC (*p* = 0.041).

### Patients with long progression-free survival

There were 43 patients who were progression-free at 5 years after allo-SCT. Their median OS was not reached and median PFS was 15.2 years (95% CI, 9.0–21.4). The characteristics of these long-term survivors are summarized in Table 5. Twenty-six (60%) of them were transplanted before 2008 and the induction therapy was AD or VAD in 23 (54%) and a thalidomide-based combination in 10 (23%) patients. Six (14%) received a bortezomib-based induction. The number of pre-allo-SCT treatment lines was 1–2 in 86%. Only 16% had ISS stage III disease and 26% extramedullary disease. All but four of these long-term survivors were transplanted early in the course of the disease. The majority had reached a good response before allo-SCT, with 18% sCR/CR and 77% VGPR/PR. The majority (72%) had chronic GVHD.

**Table 5:** Characteristics of the long-term survivors

Characteristics		Missing
*N* (%)	43 (100)	
Age, years, median (range)	50.1 (26.3–65.1)	
< 50	21 (49)	
50–60	17 (39)	
> 60	5 (12)	
Gender male/female	21/22 (49/51)	
Myeloma subtype		
IgG	24 (56)	
IgA	5 (11)	
IgD	2 (5)	
Light chain	12 (28)	
ISS stage		5 (12)
I	18 (42)	
II	13 (30)	
III	7 (16)	
Cytogeneticsa		13 (30)
G-Band analysis normal	17 (40)	
Monosomy 13	6 (14)	
14q32 abnormality	4 (9)	
Hyperdiploidy	3 (7)	
Extramedullary diseaseb	11 (26)	4 (9)
Number of pre-allo-SCT treatment lines		
1	23 (53)	
2	14 (33)	
3	5 (12)	
≥ 4	1 (2)	
Novel drugs prior to allo-SCT		
IMID	6 (14)	
PI	7 (16)	
IMID and PI	11 (26)	
None	19 (44)	
Disease status prior to allo-SCT		
sCR/CR	8 (18)	
VGPR/PR	33 (77)	
PD	2 (5)	
Time between diagnosis and allo-SCT, days, median (range)	329 (137–1543)	
Therapy group		
Upfront	19 (44)	
Auto-allo	20 (47)	
Relapse	4 (9)	
Conditioning regimen		
MACc	20 (47)	
CyTBI	16 (37)	
Treo14	4 (9.5)	
RICd	23 (53)	
FluTBI	19 (44)	
Treo-RIC	4 (9.5)	
GVHD		
Acute GVHD grades 2–4	8 (19)	
Chronic GVHD at 5 years	31 (72)	

^a^Other findings: t(11;14), del1p, trisomy 11 (each with one patient). One with del17p and one with t(4;14), both also had del(13)

^b^The presence of plasma cells or plasmacytomas outside the bone marrow

^c^MAC regimens included CyTBI (cyclophosphamide 60 mg/kg for 2 days and total body irradiation 12 Gy) and Treo14 (treosulfan 14 g/m^2^ for 3 days and fludarabine 30 mg/m^2^ for 5 days)

^d^RIC regimens included FluTBI (fludarabine 30 mg/m^2^/3 days and total body irradiation 2 Gy) and Treo-RIC (treosulfan 10–12 g/m^2^ for 3 days and flurarabine 30 mg/m^2^ for 5 days)

*ISS* International Staging System, *allo-SCT* allogeneic hematopoietic stem cell transplantation, *IMID* immunomodulatory drug, *PI* proteasome inhibitor, *sCR* stringent complete response, *CR* complete response, *VGPR* very good partial response, *PR* partial response, *MR* minimal response, *SD* stable disease, *PD* progressive disease, *Upfront-allo* allo-SCT performed first line without a previous auto-SCT, *auto-allo* allo-SCT performed after auto-SCT in first line, *relapse* allo-SCT performed after relapse, *MAC* myeloablative conditioning, *RIC* reduced-intensity conditioning, *GVHD* graft-versus-host disease

## Discussion

We have conducted allo-SCT for over 200 patients with MM, mostly (73%) as first-line therapy (upfront-allo and auto-allo). Of the three therapy groups, the auto-allo group had the longest median OS, 11.2 years. Other studies with 6- to 7-year follow-up times after auto-allo SCT have resulted in a median OS of 5.9–11.4 years [11, 15, 31].

We had 75 patients who received allo-SCT without a prior ASCT or relapse. The median OS of this group was nearly 10 years. Previous studies have reported median OS time of 1.5–3.3 years with first-line allo-SCT [1, 20]. The 5-year NRM in our upfront-allo-SCT group, with CyTBI conditioning given to 53 patients, was significantly lower than the 30–41% NRM reported in the literature [1, 20]. In fact, NRM was low overall in our study and compares well to the NRM of 10–37% reported elsewhere [5, 6, 15, 31, 32].

Compared with the OS, PFS was considerably shorter in both upfront-allo and auto-allo groups of our study. This is in line with the 0.8–4.0 years reported previously [1, 6, 11, 15, 20, 33]. The discrepancy between a long median OS but disappointingly short PFS for upfront-allo-SCT is explained by the long PRS of 4.1 years that is probably largely due to modern and more efficacious drug combinations used to treat relapse. Costa et al. performed a pooled analysis with 1338 newly diagnosed MM patients from four prospective auto-allo trials, with a median PRS of 5.2 years [34]. Others have reported PRS of 1.8–6.4 years after allo-SCT [15, 33, 35, 36]. In our study, the RI curve seemed to form a plateau at 10–15 years, indicating that 20–25% of the patients achieve long-term remission, as seen in previous studies with a long follow-up [3, 6, 8]. These findings are perhaps an indication of the importance of immunology and effective graft-versus-myeloma effect [32, 37, 38]. Also, a previous upfront allo-SCT does not seem to worsen the response to relapse treatment.

In regard to allo-SCT for relapsed/refractory MM, the outcomes were clearly inferior to the other two therapy groups. Patients relapsed early despite only four patients (7%) having PD at the time of allo-SCT. Even sCR/CR at the time of allo-SCT did not result in improved survival. The patients in the relapse group were not very heavily pretreated as 76% had received only 2–3 previous treatment lines before allo-SCT. The PRS of the relapse group was only 2.6 years. In the study by Greil et al. with retrospective analysis of 109 patients, those transplanted first line had better OS and PFS than those receiving allo-SCT in relapsed/refractory phase [6].

Our observation of aGVHD incidence of 24%, the detrimental effect of aGVHD, and the beneficial effect of cGVHD on the outcome of the patients is in line with several previous studies [12, 32, 39]. For cGVHD, the rate of 62% in our study was in the upper range compared with 27–67% reported elsewhere [16, 31, 39, 40]. This translated to the GRFS median of less than a year. However, GRFS does not evaluate cGVHD in a time-dependent manner and there may also be differences between the centers in the threshold for initiating the systemic cGVHD treatment. It should be noted that cGVHD may affect quality of life of the patients, but this data was not available to us.

We were able to define cytogenetic risk according to current criteria [21] for less than half of the patients, approximately one-third of them with HR cytogenetics. Several studies have shown that allo-SCT could overcome the adverse prognosis of high cytogenetic risk [6, 11–14], while others have not [15, 16]. In our study, allo-SCT did not seem to benefit patients with HR cytogenetics despite a majority of them receiving allo-SCT in an upfront setting. Their overall and post-relapse survival was markedly reduced compared with SR patients. The immunological effect of allo-SCT may be too slow to revert the rapid disease progression in patients with HR cytogenetics. However, our results should be interpreted with caution due to the limited number of patients.

In our study, MAC associated with a better OS than RIC, but there was no difference in PFS or RI. There were more allo-SCTs performed in relapsed/refractory MM in RIC than in the MAC group, but nevertheless, in multivariate analysis, MAC retained its independent prognostic value. In a large retrospective EBMT analysis, MAC was associated with poor OS in 1991–2002 but not after that [41]. In our study, the year of transplantation did not affect the survival outcomes.

Treosulfan-based conditioning has led to favorable responses and low NRM in some studies [5, 42], thus characterized as reduced-toxicity conditioning. In our study, 98 patients received treosulfan-based conditioning, 33 treo14, and 65 treo-RIC. Both resulted in good 5-year NRM rates of 3% and 5% but median PFS was equal to other conditioning regimens.

The serological status of myeloma before allo-SCT did not affect the survival outcomes in our study, although the proportion of patients in PD before allo-SCT was small. Even if limited by a small number of patients, it was interesting to see that in the auto-allo group, patients with sCR/CR before allo-SCT (*n* = 22) had a very long median PFS of 7.5 years (data not shown). This tendency did not show in upfront-allo group. This may be an indicator of the debulking effect of ASCT resulting in deeper response than anti-MM drugs alone.

Our study has some obvious weaknesses, including its retrospective nature and the long time span of 17 years. There has been marked development during these years in cytogenetic analyses, anti-MM drugs, and supportive care after allo-SCT. This evolution is certainly reflected in the changes in the guidelines regarding indications for allo-SCT in myeloma [17–19, 43]. Even if there did not seem to be great differences between the patients in the three therapy groups, we cannot exclude the possibility that the allocation criteria for one or another transplant strategy could have had an effect on the results. Especially during the first half of our study period, the national reimbursement policy may have affected patient referral strategy. Also, data on induction therapy response, and different post-relapse therapies, was not available to us except in the case of a DLI. As the purpose of our study was to analyze the outcome of different allo-SCT timings, we did not have an ASCT group for comparison. The referral area and post-SCT follow-up strategy of ASCT patients is different from allo-SCT patients in our center. Therefore, we would also not have been able to report the outcomes of ASCT patients with accuracy similar to allo-SCT.

In addition to allo-SCT, there are other treatment approaches with a possible chance for cure in MM, as the donor-derived chimeric antigen receptor T cell (CAR-T) or CAR-NK-cell therapy [44, 45]. These therapy forms may even replace allo-SCT in the future. A tempting approach could also be combining immunological treatment approaches [46] to allo-SCT. Adding novel agents to the conditioning regimen has shown promising results [47]. Maintenance treatment with novel agents after allo-SCT has also been studied [48–50]. Maintenance therapy should possibly be guided by measurable residual disease and donor chimerism. However, in the work by Rasche et al. [13] and Chhabra et al. [35], the majority of patients with relapse still displayed full donor chimerism in blood or bone marrow.

As a conclusion, in this single-center study with a fairly large number of patients and 5-year follow-up time, we observed relatively good outcomes in terms of long OS and low NRM in upfront-allo and auto-allo transplantations. Auto-allo-SCT as an upfront treatment seemed to be the best approach of these regimes in our material. However, achieving a long-lasting remission was challenging as PFS was relatively short. Patients with long PFS of at least 5 years were characterized by allo-SCT performed early in the course of the disease, ISS stage I and chronic GVHD. PRS was quite good, possibly due to graft-versus-myeloma effect as indicated by the beneficial effect of cGVHD and modern MM treatment. Patients with HR cytogenetics did not seem to benefit from allo-SCT. Further studies are needed to show if the relapse rate could be decreased by combining immunomodulatory drugs either to the conditioning regimen or to the post-allo-SCT period.

## Data Availability

The datasets generated and analyzed during the study are available from the corresponding author on reasonable request.
